# A Novel Foam Contrast Agent Suitable for Fluoroscopic Interventional Procedure: Comparative Study of Physical Properties and Experimental Intervention in Animal Model

**DOI:** 10.1155/2015/974537

**Published:** 2015-08-20

**Authors:** Jin Ho Hwang, Hong Suk Park, Soowon Seo, In Wook Choo, Young Soo Do, Sung Wook Choo, Sung Wook Shin, Kwang Bo Park, Sung Ki Cho, Dongho Hyun, Sooyoun Lim

**Affiliations:** ^1^Department of Radiology, Samsung Medical Center, Sungkyunkwan University School of Medicine, Seoul 135-710, Republic of Korea; ^2^Medical Device Development Center, Daegu-Gyeongbuk Medical Innovation Foundation, Daegu 701-310, Republic of Korea

## Abstract

In fluoroscopic contrast study for interventional procedure, liquid contrast agent may be diluted in body fluid, losing its contrast effect. We developed a novel contrast agent of “foam state” to maintain contrast effect for enough time and performed a comparative study of physical properties and its usefulness in experimental intervention in animal model. The mean size of microbubble of foam contrast was 13.8 ± 3.6 *µ*m. The viscosity was 201.0 ± 0.624 cP (centipoise) and the specific gravity was 0.616. The foam decayed slowly and it had 97.5 minutes of half-life. In terms of the sustainability in a slow flow environment, foam contrast washed out much more slowly than a conventional contrast. In experimental colonic stent placement, foam contrast revealed significantly better results than conventional contrast in procedure time, total amount of contrast usage, and the number of injections (*p* < 0.05). Our foam contrast has high viscosity and low specific gravity and maintains foam state for a sufficient time. Foam contrast with these properties was useful in experimental intervention in animal model. We anticipate that foam contrast may be applied to various kinds of interventional procedures.

## 1. Introduction

Fluoroscopic contrast study has been a valuable tool for diagnosis and interventional treatment of various diseases from gastrointestinal, hepatobiliary, genitourinary, musculoskeletal, and cardiovascular system [1–4]. Although overall frequency of fluoroscopic study for diagnosis has been decreasing due to the advances of cross-sectional imaging, such as computed tomography and magnetic resonance imaging, and extensive use of endoscopy, contrast study for interventional purposes is increasing continuously.

Fluoroscopic contrast agents absorb X-rays more strongly than the organ being examined and appear radiopaque on fluoroscopy. When they are introduced into hollow viscus or tubular structure, they visualize anatomy as well as pathology. They are barium sulfate suspension and iodine ionic or nonionic compounds, and all in liquid forms [1]. Due to lack of specialized contrast agents, most fluoroscopic interventional procedures are performed using the same conventional “liquid state” contrast agents before, during, and after the procedure.

Conventional liquid contrast agents may be easily dispersed and diluted in the body fluid or blood, rapidly losing its contrast effect. Repeated injections are required during intervention, causing an increase in the amount of contrast agent and prolongation of fluoroscopic time.

We developed a novel contrast agent of foam state made from human serum albumin solution, room air, and iodinated contrast powder. In this study, we tested its physical properties and evaluated that its rheological properties would be helpful in experimental interventional procedure in animal model.

## 2. Methods

The foam contrast was made from human serum albumin 20% solution (SK Chemicals. Co. Ltd., Korea) mixed with iodine contrast powder and room air. First, we dissolved the contrast powder (612 mg) per 1 mL of albumin solution to prepare a total of 4 mL mixed solution. Then, we mixed that solution with the same amount (4 mL) of room air by the pumping method (Tessari's method). Two syringes were attached using a three-way stopcock, and a stable foam contrast was created by mixing them through multiple passages (20–30 times) between the two syringes.

### 2.1. Physical Property

#### 2.1.1. Foam Size

We measured the size of microbubble of foam contrast through an optical microscope. Differences of microbubble size caused by changing temperature and pH were also examined.

#### 2.1.2. Viscosity and Specific Gravity

The viscosity and specific gravity of foam contrast were measured using viscometer (HBDV-II + Pro, Brookfield Engineering Laboratories, Inc., MA, USA) and electronic densitometer (MD-300S, Alfa Mirage Co., Ltd., Osaka, Japan) at ambient temperature of 23°C by the external testing laboratory (Korea Polymer Testing & Research Institute, Seoul, Korea).

#### 2.1.3. CT Hounsfield Numbers

To compare the visual difference of foam contrast agent and conventional contrast media (Ultravist 300; Schering AG, Berlin, Germany), we measured CT Hounsfield unit (HU) of pure and half diluted conventional contrast agent and foam contrast using a CT scanner (Inveon CT; Siemens Healthcare, Erlangen, Germany).

#### 2.1.4. Foam Decay and Sustainability

In terms of foam persistence, we measured the remaining amount of the foam content at 1, 2, 3, and 5 minutes and then every 5 minutes up to 120 minutes. We estimated the half-life of foam contrast agent, which is time required for half of the entities to decay or disintegrate.

**Figure 1:**
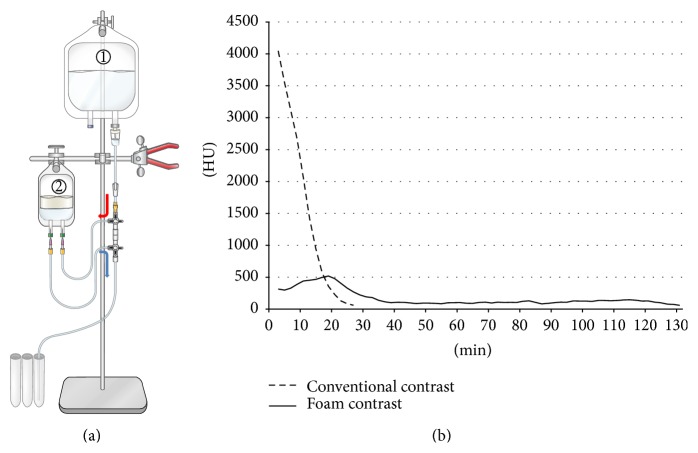
(a) An experimental model to examine the sustainability of contrast agents in a slow flow environment. Normal saline (①) is infused into the bag (②) filled with foam contrast or conventional contrast, and the outflow was collected every 2 minutes. (b) Results of the sustainability of contrast effect. CT numbers of collected outflow from conventional contrast (dotted line) and foam contrast (solid line) were plotted over time. Note the rapid decrease in CT number of conventional contrast and sluggish curve of foam contrast agent.

Sustainability of foam contrast in an experimental model with slow flow (Figure 1(a)) was tested and compared between foam contrast agent and conventional contrast media, as follows: 30 mL of foam contrast or conventional contrast was filled in a 100 mL transparent plastic infusion bag. 500 mL of normal saline was infused into this bag and simultaneously, the outflow was collected every 2 minutes and the CT HU were measured, respectively. Hence, we estimated the sustainability of contrast effect indirectly.

### 2.2. Animal Study

#### 2.2.1. Animal Model

We developed and reported an animal model of colonic obstruction for the development and evaluation of gastrointestinal stent [1]. Briefly, a surgical obstruction model was made in healthy mongrel dogs, as follows. The dogs, weighing 19.9–28.5 kg (mean, 23.3 kg), were acclimated and individually housed for 7 days before experiments. After general anesthesia, a segment of the descending colon was exposed after a lower midline incision and was wrapped with a nonabsorbable synthetic mesh (Prolene Mesh, Ethicon, Inc., Somerville, NJ, USA) of a proper length. The mesh was punched to make four holes at each end and four flat rubber bands were passed through these holes in the mesh and the mesentery. The rubber bands were tightened to induce the complete obliteration of the colonic lumen and fixed with contact adhesives. Surgical suture material was used to put together the mesh and rubber bands and to fix them to the colonic wall. The abdomen was closed surgically.

This study was approved by the Institutional Animal Care and Use Committee, and all the procedures were conducted in accordance with the eighth edition of the Guide for the Care and Use of Laboratory Animals published by National Research Council of the National Academies, 2011, and followed the guidelines of Samsung Biomedical Research Institute (Seoul, Korea), which has been accredited by the Association for Assessment and Accreditation of Laboratory Animal Care (AAALAC) International.

#### 2.2.2. Experimental Fluoroscopy-Guided Gastrointestinal Stent Placement

On the fourth day after laparotomy, dogs were randomly assigned to insert the stents under fluoroscopic contrast study using foam contrast agent in the study group (*n* = 5) and conventional contrast media in the control group (*n* = 5) via anal route with a 5 Fr catheter, under general anesthesia.

A 5 Fr angiographic catheter and a 260-cm-long, 0.035-inch-diameter hydrophilic guide wire were passed through the obstructed segment with the aid of fluoroscopic contrast study using either foam contrast or conventional contrast. Self-expandable covered metallic stents (Bonastent, Standard Sci-Tech Inc., Seoul, Korea) were placed in the obstructed segments of the colon along the guide wire. After the stent was deployed, a contrast study was obtained to verify the position and patency of the stent.

We measured the time of procedure, total amount of contrast usage, and the number of injections during the stenting procedure.

Statistical analysis were performed using SPSS software version 20 (IBM Inc., Chicago, Il, USA). Mann-Whitney* U* test was used to compare the results of both groups in animal study and *p* value < 0.05 was considered significant.

## 3. Results

### 3.1. Physical Properties

**Figure 2:**
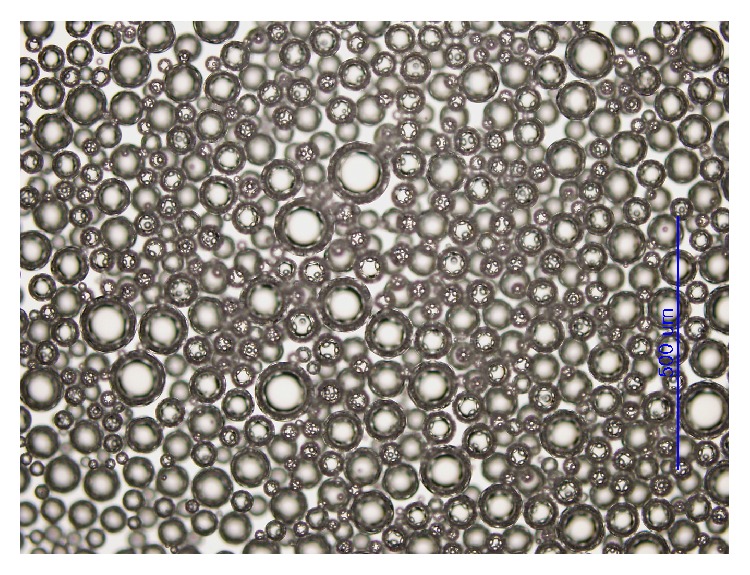
Optical microscope of microbubbles in foam contrast agent.

The size of the microbubble of foam contrast was 13.8 ± 3.6 *μ*m (Figure 2) and the smallest in lower temperature and in neutral pH. The viscosity of foam contrast was 201.0 ± 0.624 cP (centipoise) and the specific gravity of foam contrast was 0.616. The value of CT HU of foam contrast was 3023 HU, and pure and half diluted conventional contrast were 4281 HU and 3116 HU, respectively.

**Figure 3:**
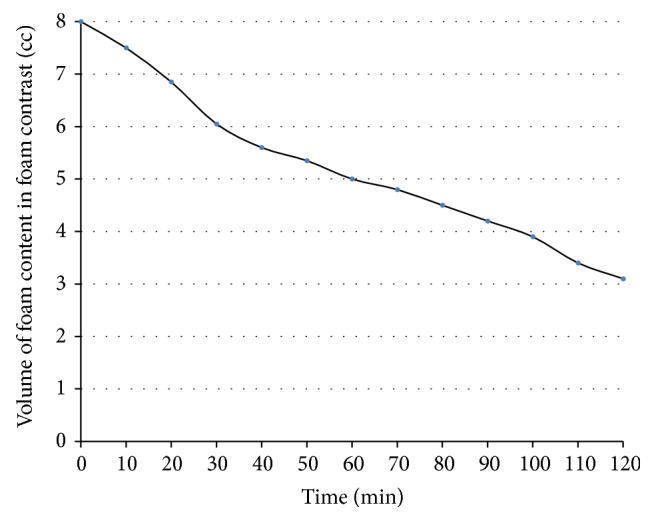
Foam decay with time. The half-life was 97.5 minutes.

The foam decayed with time showing a linear decrease and the half-life of foam contrast (Figure 3) was approximately 97.5 minutes.

In a slow flow experiment to examine the sustainability of contrast agents (Figure 1(b)), CT HU of the outflow of conventional contrast media was the highest level at the initial measurement and then decreased rapidly within 26 minutes. By contrast, CT HU of the outflow of foam contrast agent was the highest level at 18 minutes and decreased slowly and reached a plateau that lasted about 100 minutes.

### 3.2. Stent Placement Using Foam and Conventional Contrast Agent

**Figure 4:**
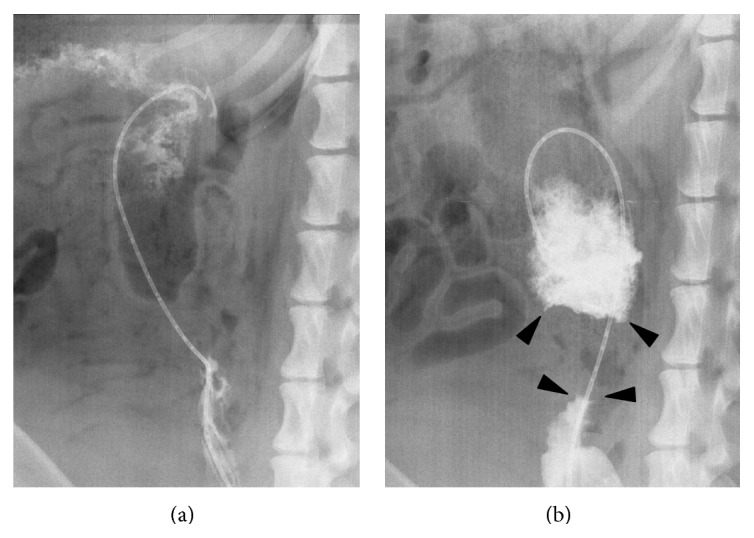
Fluoroscopic contrast study showing colonic obstructed segment using conventional contrast agent (a) and foam contrast agent (b). In contrast with conventional contrast agent that flowed downward easily, fluoroscopic contrast study with foam contrast visualized the obstructive lesion (arrowheads) clearly.

Stent placement was successful without any complication in all dogs (Figure 4). Procedure time was 12.2 ± 4.0 min in the study group (foam contrast), and 22.0 ± 8.8 min in the control group (*p* = 0.047). Total amount of contrast usage was 6.0 ± 2.2 cc in the study group, and 28.0 ± 7.6 cc in the control group (*p* = 0.007). The number of injections of contrast agent during the procedure was 1.2 ± 0.4 in the study group, and 5.6 ± 1.5 in the control group (*p* = 0.007).

## 4. Discussion

During interventional procedures using fluoroscopic guidance, the visualization of lesions is essential to the placement of the interventional instruments, such as wire, catheter, balloon, or stent, in proper position. For vascular lesions, we may use roadmap angiography, which is a real-time fluoroscopic image overlaid on a static digitally subtracted angiographic images of blood vessels. However, roadmapping cannot be used in the intervention of slow flow environment, such as gastrointestinal or biliary tract. Conventional fluoroscopic contrast study should be used and repeated injection of contrast agent may be needed. It would be bothersome and time-consuming, resulting in elongation of procedure time and increasing radiation exposure.

Stent placement is established as an effective palliation for benign and malignant gastrointestinal obstruction [5–12] and can be performed using either an endoscopic method or a radiologic method using fluoroscopy, or both. During the procedure using fluoroscopy, contrast media should be used to depict the stricture. The duration of the procedure is highly variable and dependent on the degree of difficulty in accessing or traversing the stricture [10, 13, 14]. Therefore, depicting the obstructed segment for enough time is important in the stenting procedure. During the previous animal study of colonic stent placement [15], we experienced this problem; the anal sphincteric tone of the animal under general anesthesia was absent and injected contrast agent easily flew out, resulting in poor visualization of the obstructing lesion. This occurs frequently in human patients in poor general condition. Therefore, we developed a novel contrast of foam state to solve this problem. Foam agent has high viscosity and low specific gravity, thus allowing the adhering of contrast agent to the wall of target lesion relatively unaffected by gravity. Foam agent also has malleability and does not easily diffuse or get diluted in liquid. These rheological properties show positive effects that contrast agent of foam state will visualize complex anatomy and will have sustained contrast effect even in secretion or fluid of food or fecal material.

Foam sclerosing agents, which were first introduced for the treatment of venous diseases in 1939 by Stuard McAusland [16], are the most widely used foam material in the clinical field. According to reported literatures, the rationale for the use of foam sclerosing agents was replacement of blood by the sclerosing foams and to ensure that the sclerosant may get in close contact with the venous intima [16–18]. There have been many techniques to prepare foam agent, and the most commonly used technique is Tessari's method [19]. Two syringes are connected through a three-way stopcock, and liquid agent and air are drawn back and forth by pumping movement. Medically relevant microbubbles are stabilized by encapsulating gas within shells that comprised protein, protein plus sugar, lipids, polymers, or combinations of these materials [20]. We applied this concept to our new foam contrast using the simple and easy Tessari's method and prepared human serum albumin acting as the protein shell and room air as encapsulated gas.

Foam is a state of high energy and thus so unstable that it may decay with time. Therefore, persistence of foam is very important for practical use. Proteins are the most widespread foam stabilizers used in food manufacturing [21]. We used human serum albumin as a foam stabilizer and thus we could make foam contrast with a long-lasting foam state.

Conventional contrast agent has viscosity of 4.6 cP, does not adhere to the intestinal mucosa, and easily flows down along the direction of gravity. Moreover, peristaltic movement of gastrointestinal tract also does much for flowing down of contrast media. To overcome these problems, operators need to inject contrast during the interventional procedure frequently and the total amount of contrast media and the procedure time would increase accordingly. Viscosity of our novel foam contrast was 201.0 ± 0.624 cP, much higher than that of conventional contrast agents. This elongated the contrast effect and thus decreased the usage of contrast agent and the procedure time in our animal study.

Foam has another important characteristic, which is that it does not easily mix with adjacent fluid and rather aggregates together. Due to this characteristic, along with high viscosity and low specific gravity, foam contrast did not easily wash out and sustained foam state and contrast effect in our slow flow experimental model and animal study.

Foam contrast can be effective in fluoroscopic contrast study during sclerotherapy using foam sclerosing agent, such as balloon-occluded retrograde transvenous obliteration (BRTO) of gastric varices in liver cirrhosis, embolization of varicocele or varicose vein, or sclerotherapy of congenital venous malformation [22–25]. In these procedures, venography with conventional contrast is performed to delineate the lesion and to determine the amount of sclerosing agent and then foam sclerosing agent is introduced. Because of a significant difference in specific gravity between conventional contrast and foam sclerosing agent, foam sclerosing agent in blood-filled dilated venous lesion tends to distribute differently from the contrast dose. In this situation, foam contrast can visualize the lesion in advance, distributing similarly to foam sclerosing agent and this will enable an accurate decision of treatment strategies and amount of sclerosing agent.

Our study has some limitations. First, the number of dogs used in the experiments was small although there was statistical power. Second, since albumin solution is expensive, we need further investigation of an inexpensive and harmless foam stabilizer with prolonged foam state, such as nonionic surfactant, for practical utilization. Further prospective and comparative clinical studies with human patients and a longer follow-up are needed to reproduce these results before foam contrast can be recommended. The CT number of foam contrast (3023 HU) was lower than that of pure contrast (4281 HU) and similar to that of half diluted conventional contrast. There was no practical difference of contrast effect between foam contrast and conventional contrast under fluoroscopy during our animal study.

In conclusion, our novel foam contrast agent has high viscosity and low specific gravity compared with conventional contrast agents and maintains foam state for a sufficient period of time. Due to these characteristics, we could reduce usage of contrast and procedure time in our animal study and we anticipate that foam contrast may be helpful in fluoroscopic contrast study during various kinds of interventional procedures.
